# Identification and significance of *Weissella* species infections

**DOI:** 10.3389/fmicb.2015.01204

**Published:** 2015-10-31

**Authors:** Kamal Kamboj, Amber Vasquez, Joan-Miquel Balada-Llasat

**Affiliations:** ^1^Clinical Microbiology Laboratory, Department of Pathology, The Ohio State University Wexner Medical CenterColumbus, OH, USA; ^2^Department of Internal Medicine, Division of Infectious Diseases, The Ohio State University Wexner Medical CenterColumbus, OH, USA

**Keywords:** *Lactobacillus* spp., *Weissella* species, vancomycin resistance, MALDI-TOF MS

## Abstract

*Weissella* spp. are non-spore forming, catalase-negative, gram-positive coccobacilli. They are often misidentified by traditional and commercial phenotypic identification methods as *Lactobacillus* spp. or *Lactobacillu*s-like organisms. *Weissella* spp. were previously grouped along with *Lactobacillus* spp., *Leuconostoc* spp., and *Pediococcus* spp. Utilization of more sensitive methods like DNA sequencing or Matrix-Assisted Laser Desorption/Ionization Time of Flight Mass Spectrometry (MALDI-TOF MS) has facilitated identification of *Weissella* as a unique genus. Nineteen species have been identified to date. *W. confusa, W. cibaria*, and *W. viridescens* are the only species isolated from humans. The true prevalence of *Weissella* spp. continues to be probably underestimated. *Weissella* spp. strains have been isolated from a wide range of habitats including raw milk, feces, fermented cereals, and vegetables. *Weisella* is believed to be a rare cause of usually nonfatal infections in humans, and is often considered a contaminant. However, in recent years, *Weissella* spp. have been implicated in bacteremia, abscesses, prosthetic joint infections, and infective endocarditis. Alterations of the gut flora from surgery or chemotherapy are believed to facilitate translocation of *Weissella* spp. due to disruption of the mucosal barrier, predisposing the host to infection with this organism. Implications of the isolation of *Weissella* spp. from blood must be interpreted in context of underlying risk factors. *Weissella* spp. are inherently resistant to vancomycin. Therefore, early consideraton of the pathogenic role of this bacteria and choice of alternate therapy is important to assure better outcomes.

## Introduction

*Weissella* spp. are non-spore forming, hetero-fermentative, facultative anaerobic, gram-positive, catalase-negative, alpha hemolytic bacteria that appear as short rods or coccobacilli in pairs and chains (Collins et al., 1993; Olano et al., 2001; Björkroth et al., 2009). Based on their unusual Gram stain morphology and inherent resistance to vancomycin, *Weissella* spp. have been often confused with *Lactobacillus* spp. or *Lactobacillu*s-like organisms. They are usually considered contaminants when recovered from clinical specimens and rarely identified to the species level due to their fastidious nature (Facklam et al., 1989; Facklam and Elliott, 1995; Kumar et al., 2011).

*Weissella* was identified as a unique genus in 1993 on the basis of 16S rRNA gene sequence analysis and named after Norbert Weiss, a German microbiologist, for his many contributions to the taxonomy of lactic acid bacteria (Collins et al., 1993). *Leuconostoc paramesenteroides* and related species amongst the catalase-negative, vancomycin-resistant, gram-positive cocci were reclassified into this genus. *Weissella* now constitute a distinct phylogenetic group, separate from those of other genera of lactic acid bacteria, including *Leuconostoc, Lactobacillus*, and *Streptococcus* (Flaherty et al., 2003). To date, nineteen species of *Weissella* have been identified, namely: *W. beninensis, W. ceti, W cibaria, W. confusa, W. diestrammenae, W. fabalis, W. fabaria, W. ghanensis, W. halotolerans, W. hellenica, W. kandleri, W. koreensis, W. minor, W. oryzae, W. paramesenteroides, W. soli, W. thailandensis, W. uvarum, W. viridescens* (Fusco et al., 2015). All species except *W. beninensis* are non-motile (Padonou et al., 2010; Björkroth et al., 2014). Of these, only *W. confus*a (previously *Lactobacillus confusus*), *W. cibaria*, and *W. viridescens* have been isolated from human clinical specimens (Björkroth et al., 2002; Kulwichit et al., 2007; Fusco et al., 2015) and considered as opportunistic pathogens (Fusco et al., 2015). *W. confusa* has also been documented as a cause of systemic infection in a healthy primate (*Cercopitheus mona*; Vela et al., 2003) and neonatal sepsis in a foal (Lawhon et al., 2014). *W. confusa* and *W. cibaria* both have the ability to produce NH_3_ from arginine but differ in their ability to acidify different sugars (Björkroth et al., 2002). Unlike *W. confusa, W. cibara* is negative for the fermentation of galactose and xylose and positive for the fermentation of arabinose (Björkroth et al., 2002). Several species of *Weissella*, including some strains of *W. confusa*, can grow at 25, 35, and 45°C (Olano et al., 2001; Vasquez et al., 2015). *Weissella* species have been used in the production of a variety of fermented foods and beverages and also as probiotics (Kang et al., 2011; Lee et al., 2012; Gomathi et al., 2014; Zhang et al., 2014). *W. cibaria* possesses anti-cancer, anti-inflammatory, antibacterial, anti-fungal, and immune boosting potential (Nam et al., 2002; Kang et al., 2006; Srionnual et al., 2007; Lee et al., 2008, 2013; Valerio et al., 2009; Ahn et al., 2013; Kwak et al., 2014).

## Epidemiology

*Weissella* spp. have a widespread distribution and have been isolated from a wide variety of habitats including raw milk, feces, saliva, breast milk, urine, fermented cereals, meat and meat products, sugar cane, carrot juice, banana leaves and vegetables (Kandler and Weiss, 1986; Björkroth et al., 2002; Fairfax et al., 2014; Fusco et al., 2015). They have also been recovered from feces of healthy individuals (Green et al., 1990; Walter et al., 2001) and are common inhabitants of the vaginal microbiota (Jin et al., 2007). Although *Weissella* are of rare occurrence in humans, several disease outbreaks involving *W. ceti* have been reported in cultured rainbow trout from geographically diverse locations (United States, China, Brazil; Liu et al., 2009; Figueiredo et al., 2012; Welch and Good, 2013; Costa et al., 2015). The true incidence of *Weissella* infection in humans is probably underestimated due to its misidentification.

## Diagnosis

Identification of *Weissella* at the genus and species level has been challenging. It is often misidentified as *Lactobacillus*-like or viridans streptococci and accurate identification is not possible by traditional or commercial phenotypic identification methods that include morphological analysis, growth characteristics and sugar fermentation profiles as these techniques have low taxonomic discrimination (Fusco et al., 2015). Commercial biochemical based identification kits namely API (Analytical Profile Index systems, bioMéreiux, France) and RapiID Strep panel (Remel, USA) are unable to identify *Weissella* species. The identification table of API 20 Strep does not include *Weissella*. API 50 CHL kit (version 5.1) has *W. confusa* and *W. viridescens* in its identification table; however, it cannot differentiate *W. cibara* from *W. confusa* (Kulwichit et al., 2007). RapID™ STR System does include *W. confusa* in its database but only give a high probability result (Fairfax et al., 2014). Automated systems namely Vitek 2 (bioMéreiux, France), MicroScan (Beckman Coulter Inc. USA), and Phoenix Automated Microbiology System (BD Diagnostic Systems, USA), do not have *Weissella* in their database and as such cannot reliably identify *Weissella* species (Lee et al., 2011; Fairfax et al., 2014; Fusco et al., 2015).

Molecular DNA sequencing involving 16S rRNA gene sequence analysis can accurately identify *Weissella* to the species level and remains the current gold standard. It has also emerged as powerful tool for identification of phenotypically atypical microorganisms (Petti et al., 2005) and has been successfully used to identify *Weissella* to the species level (Collins et al., 1993; Vasquez et al., 2015). Most of the cases of *W. confusa* reported were originally misidentified as *Lactobacillus*-like or viridians streptococci organisms by phenotypic methods. These were subsequently confirmed to be *W. confusa* using 16S rRNA gene sequence analysis. Amplified ribosomal DNA restriction analysis (ARDRA; Jang et al., 2002) and ribotyping (Björkroth et al., 2002) have also been used to correctly identify *Weissella* species. Molecular typing techniques for *Weissella* species include DNA finger printing and restriction of ribosomal DNA (Villani et al., 1997), numerical analysis of *Hind*III and *Eco*RI ribopatterns (Koort et al., 2006), repetitive element-PCR fingerprinting using (GTG)_5_-PCR (Bounaix et al., 2010), and fluorescent- amplified fragment length polymorphism (fAFLP; Fusco et al., 2011). MALDI-TOF MS is now being routinely used for the identification of bacterial organisms (Bizzini et al., 2010; Wieser et al., 2012) and can also accurately identify *W. confusa* (Lee et al., 2013; Fairfax et al., 2014). Two MALDI-TOF MS systems have found increasing use in microbiology laboratories and both are sensitive for the identification of unusual and/or difficult-to-identify microorganisms isolated from clinical specimens (McElvania TeKippe and Burnham, 2014). The Bruker Biotyper (Bruker Daltonics, Germany) software version 3.0 also includes *W. confusa, W. halotolerans, W. minor*, and *W. viridescens*, and VITEK MS (bioMérieux, France) database v2.0 has *W. confusa* and *W. viridescens*. The future versions of both these mass spectrometry systems are likely to incorporate other *Weissella* spp. which will facilitate their early identification and provide insight into the true prevalence of these infections.

## Predisposing factors and clinical manifestations

Most of the cases of *W. confusa* infections reported in humans have been from immunocompromised patients (Lahtinen et al., 2012; Fairfax et al., 2014; Medford et al., 2014). Malignancy has been the most common factor associated with immunocompromised and complicated medical status. Recent chemotherapy, organ transplant, burn, chronic alcoholism, long-term use of steroids, chronic renal insufficiency and diabetes seem to increase the chances of acquiring this infection (Flaherty et al., 2003; Salimnia et al., 2011; Table 1). Orthopedic procedures like joint replacements, arthroplasty, and post-operative osteomyelitis also put the patients at increased risk of bacterial infections. Prior exposure to vancomycin may results in the selection of *Weissella*, which is intrinsically resistant to this drug. Central line catheter insertion prior to any surgical procedure increases the risk of infection, although *Weissella* has not been recovered from catheter tips. Total parenteral nutrition has been suspected to be risk factor for the development of bacteremia involving *Weissella* species (Olano et al., 2001; Flaherty et al., 2003; Kumar et al., 2011; Lee et al., 2011; Vasquez et al., 2015). *Weissella* is a common inhabitant of the human gastrointestinal system. A compromise of the gastrointestinal mucosal barrier due to surgery is associated with increased risk of acquiring this infection and may be a probable route of entry of *W. confusa* resulting in bacteremia and endocarditis (Flaherty et al., 2003; Shin et al., 2007). Polymicrobial infection and subsequent antimicrobial measures resulting in the alteration of gut flora also favor the selection of *Weissella* in such patients (Kumar et al., 2011). Changes in the normal flora of the throat, gut, and vaginal tract and disruption of mucous integrity by invasive procedures, surgery, and/or antibiotics predispose the host to increased risk of *Weissella* infection. The possible risk factors for *W. cibara* and *W. viridescens* infection in humans remain unknown.

**Table 1:** **Summary of previously documented ***Weissella confusa*** infections**.

**Age (year, sex)**	**Clinical infection**	**Underlying conditions**	Treatment^**^	**Survival**	**References**
71, M	Peritoneal fluid	Hemicolectomy	Cephalosporin	Survived	Riebel and Washington, 1990
12, F	Abdominal fluid	Gastrostomy	Cephalosporin	Survived	Riebel and Washington, 1990
49, M	Thumb abscess	Palm tree splinter in thumb	CEF	Survived	Bantar et al., 1991
46, M	Bacteremia	Abdominal surgery, polymicrobial infection	PIP-TAZ, VAN/GENT	Survived	Olano et al., 2001
49, M	Endocarditis	Alcoholism, previous steroid use, carious teeth	None	Died	Flaherty et al., 2003
65, M	Endocarditis	Aortic insufficiency	PEN, GENT, MXF, CEF	Survived	Shin et al., 2007
Unknown, F	Osteomyelitis	Surgery, bone grafting of mandible	AMP-SUL	Unknown	Kulwichit et al., 2008
4, M	Bacteremia	Peritoneal neuroblastoma, CT, ileus surgery	MEM, AZT, CXT, MTZ, TEC	Survived	Svec et al., 2007
Multiple cases^*^	Bacteremia	Malignancy (4), CT (3), chronic steroid use (3), abdominal surgery (4), polymicrobial infection (5), central catheter (6)	Miscellaneous^**^	Survived (4), Died (6)	Lee et al., 2011
34, M	Bacteremia	ALL, ASCT	VAN, AZT then DAP	Survived	Salimnia et al., 2011
52, M	Bacteremia	Severe burns, polymicrobial infection, central catheter	VAN, IPM then DAP	Survived	Salimnia et al., 2011
54, M	Bacteremia	HCC/liver transplant, diabetes	VAN, PIP-TAZ then CTX/LVX, MTZ	Survived	Harlan et al., 2011
48, M	Bacteremia	Gastro-esophageal adenocarcinoma, CT, Endoscopy	CFP- SUL, MTZ	Survived	Kumar et al., 2011
60, F	Bacteremia	Intramural hematoma of the aorta	CTX then TEI and PIP-TAZ	Survived	Lee et al., 2013
94, F	Prosthetic joint	Osteoarthritis, total knee arthroplasty	LVX	Survived	Medford et al., 2014
63, F	Bacteremia	Multiple abdominal surgeries, central catheter	DAP	Survived	Vasquez et al., 2015

*HCC, hepatocellular carcinoma; ALL, acute lymphocytic leukemia; ASCT, autologous stem cell transplant; AHSCT, autologous hematopoietic stem cell transplant; CT, chemotherapy; TPN, total parenteral nutrition*.

*AMP, ampicillin; AZT, aztreonam; CEF, cephalothin; CFP, cefoperazone; CTX, ceftriaxone; CXT, cefoxitin; DAP, daptomycin; GENT, gentamicin; IPM, imipenem; LVX, levofloxacin; MEM, meropenem; MXF, moxifloxacin; MTZ, metronidazole; PEN, penicillin; PIP-TAZ, piperacillin-tazobactam; SUL, sulbactam; TEC, teicoplanin; VAN, vancomycin*.

*M, male; F, female*.

* *6 females and 4 males with average age of 56.6 years*.

** *Ampicillin-sulbactam (2), amoxicillin –clavulanate (3), ceftazidime (3), cefepime (1), combined therapy with trimethoprim/sulfamethoxazole, vancomycin, ciprofloxacin & ceftazidime (1). After empiric therapy, antibiotics were adjusted to ampicillin-sulbactam (2), piperacillin-sulbactam (1), amoxicillin-clavulanate (1), piperacillin-tazobactam (1), penicillin (1). One patient did not receive any antibiotic (1)*.

*W. confusa* has been associated with a variety of clinical manifestations in humans (Table 1). The majority of the cases are seen in the settings of polymicrobial infections (Green et al., 1990, 1991; Bantar et al., 1991; Salimnia et al., 2011). However, it has also been recovered as the sole microbial agent in certain cases (Flaherty et al., 2003; Lee et al., 2011). Bacteremia is the major manifestation of *W. confusa* infection in humans (Olano et al., 2001; Kulwichit et al., 2007; Svec et al., 2007; Harlan et al., 2011; Lee et al., 2011, 2013; Salimnia et al., 2011; Vasquez et al., 2015). Other clinical vignettes in which this organism has been reported include endocarditis (Flaherty et al., 2003; Shin et al., 2007), post-operative osteomyelitis (Kulwichit et al., 2008), and abscess (Bantar et al., 1991). It has also been recovered from peritoneal fluid and the abdominal wall (Riebel and Washington, 1990) and infected prosthetic joint (Medford et al., 2014).

*W. cibaria* have been identified in the urine, lung, and blood of patients with bacteremia. *W. viridescens* has been recovered from human blood (Kulwichit et al., 2007) and fecal DNA from children with celiac disease (Sanz et al., 2007). The clinical significance of infections with these species is not yet clear.

## Antimicrobial susceptibility testing

*Weissella* is known to be intrinsically resistant to vancomycin and has high minimum inhibitory concentration (MIC) of ≥256 μg/ml. Antimicrobial susceptibilities of *W. confusa* are not fully understood. There are no standard methods and interpretation criteria of antimicrobial susceptibilities established so far for *Weissella* spp. by the Clinical and Laboratory Standards Institute (CLSI). Susceptibility testing methods have included broth dilution, agar based methods (Bantar et al., 1991; Olano et al., 2001; Vay et al., 2007; Lee et al., 2011; Medford et al., 2014) and *E*-test (Svec et al., 2007; Table 2). Low MICs have been noted for penicillin, ampicillin, tetracycline, clindamycin, erythromycin, ciprofloxacin, daptomycin, imipenem, fluoroquinolones (levofloxacin, moxifloxacin), amoxicillin-clavulanate, ampicillin-sulbactam, piperacillin-tazobactam, and doripenem. *W. confusa* exhibits a high level of resistance to ceftazidime, cotrimoxazole, rifampin, metronidazole, teicoplanin and trimethoprim/sulfamethoxazol.

**Table 2:** **Minimum Inhibitory Concentrations (MICs, in μg/mL) of ***Weissella confusa*** to various antibiotics**.

**Antibiotic**	**Bantar et al., 1991*n* = 1**	**Olano et al., 2001*n* = 1**	**Vay et al., 2007*n* = 2**	**Svec et al., 2007*n* = 1**	**Lee et al., 2011*n* = 10**	**Medford et al., 2014*n* = 2**
Amikacin	–	–	8	–	–	–
Amoxicillin-clavulanate	–	–	–	–	0.5–8/0.25–4	–
Amoxicillin	–	1	–	–	–	–
Ampicillin	0.5	–	0.5	0.5	0.5–1	0.5
Ampicillin-sulbactam	–	–	–	–	8–16/2–4	–
Azithromycin	–	–	–	–	0.12	–
Cefotaxime	4	–	–	3	–	–
Cefoxitin	32	–	–	–	–	–
Ceftazidime	16	–	–	–	≥128	–
Ceftobiprole	–	–	–	–	0.5–2	–
Ceftriaxone	–	4	16–64	–	–	–
Cefuroxime	8	4	8	–	–	–
Cephalothin	8	–	8	–	–	–
Chloramphenicol	8	–	1	–	–	4–8
Ciprofloxacin	1	–	8	0.5	–	≤1–2
Clindamycin	–	≤0.1	–	0.06	–	≤0.5
Cotrimoxazole	–	–	–	>32	–	–
Daptomycin	–	–	–	–	0.03–0.12	≤0.5
Doripenem	–	–	–	–	0.5–16	–
Erythromycin	≤0.125	≤0.12	0.032–0.063	0.13	–	≤0.25
Gatifloxacin	–	–	0.5–1	–	–	–
Gentamycin	1	–	8	3	–	≤0.2
Imipenem	≤0.125	–	0.125	0.06	–	–
Levofloxacin	–	–	4–8	–	–	2
Linezolid	–	–	2–4	–	2–4	2–4
Meropenem	–	–	–	–	1–16	–
Metronidazole	–	–	–	>256	–	–
Moxifloxacin	–	–	–		0.25–0.5	0.5
Penicillin	1	0.5	0.25	0.38	–	0.5
Piperacillin	–	–	4	–	–	–
Piperacillin-tazobactam	–	–	–	–	4–8/4	–
Rifampin	>8	–	64	–	–	–
Tigecycline	–	–	–	–	0.03–0.12	–
Teicoplanin	–	–	≥512	>256	–	–
Tetracycline	–	4	–	–	–	4–8
Trimethoprim-sulfamethoxazole	>64	>4	128–256	–	16–≥128	>4
Vancomycin	>256	>16	≥512	>256	>64	Resistant

## Conclusion

*W. confusa* is an opportunistic bacterial organism that warrants rapid and accurate identification to ensure appropriate therapy. It is difficult to distinguish *Weissella* species from other heterofermentative bacteria on the basis of phenotypic or biochemical tests alone. Accurate and rapid identification to the species level is feasible using 16S rRNA or MALDI-TOF MS techniques. Infections with *Weissella* spp. primarily occur in immunocompromised status and/or those with other underlying medical conditions. It is a common inhabitant of the gut flora, therefore, surgical procedures may translocate this organism and result in bacteremia, endocarditis, and abscess formation. The clinical significance of *W. confusa* remains unclear in the setting of polymicrobial infections, which comprise the largest proportion of total cases. The use of antimicrobial agents, especially vancomycin, may predispose patients to increased risk of infection with *Weissella*, which is intrinsically resistant to this drug (Lahtinen et al., 2012; Fairfax et al., 2014; Medford et al., 2014). When isolated in blood culture, *Weissella* may be confused with *Lactobacillus*-like or viridians streptococci and can be considered a probable contaminant (Ruoff, 2002; Fairfax and Salimnia, 2012; Fairfax et al., 2014). However, *Weissella* is not a part of the normal skin flora and should be considered significant when isolated from blood cultures (Petti et al., 2005).

Vancomycin is the empiric first-line therapy for bacteremia caused by gram-positive organisms. However, the use of vancomycin is likely to favor the growth of vancomycin-resistant organisms and may predispose these patients to subsequent infections with *Weissella* and other vancomycin resistant organisms (Fairfax and Salimnia, 2012). Early consideration of this organism in the differential diagnosis is important due to its inherent vancomycin resistance, which necessitates alternative therapy. Management of positive blood cultures especially from immunocompromised patients is a challenge both for the laboratory microbiologists as well as the clinicians. Penicillin, ampicillin, imipenem, clindamycin, erythromycin, moxifloxacin, doripenem, daptomycin, and tigecycline are all therapeutic agents for treating *Weissella* infections. The use of vancomycin, metronidazole, rifampin, teicoplanin, ceftazadime, and trimethoprim-sulphamethoxazole should be avoided if *Weissella* spp. is suspected. Antimicrobial susceptibility testing is vital to guide appropriate therapy in cases of severe infections.

### Conflict of interest statement

The authors declare that the research was conducted in the absence of any commercial or financial relationships that could be construed as a potential conflict of interest.
